# Oxidative Stress, Reductive Stress and Antioxidants in Vascular Pathogenesis and Aging

**DOI:** 10.3390/antiox12051126

**Published:** 2023-05-19

**Authors:** Mitko Mladenov, Lubomir Lubomirov, Olaf Grisk, Dimiter Avtanski, Vadim Mitrokhin, Iliyana Sazdova, Milena Keremidarska-Markova, Yana Danailova, Georgi Nikolaev, Rossitza Konakchieva, Hristo Gagov

**Affiliations:** 1 Faculty of Natural Sciences and Mathematics, Institute of Biology, “Ss. Cyril and Methodius” University, P.O. Box 162, 1000 Skopje, North Macedonia; mitkom@pmf.ukim.mk; 2 Institute of Physiology, Brandenburg Medical School Theodor Fontane, 16816 Neuruppin, Germany; lubomir.lubomirov@mhb-fontane.de (L.L.); olaf.grisk@mhb-fontane.de (O.G.); 3 Friedman Diabetes Institute, Lenox Hill Hospital, Northwell Health, 110 E 59th Street, New York, NY 10003, USA; davtanski@northwell.edu; 4 Department of Physiology, Pirogov Russian National Research Medical University, 1 Ostrovityanova Street, 117997 Moscow, Russia; mitrokhin_vm@rsmu.ru; 5 Department of Animal and Human Physiology, Faculty of Biology, Sofia University “St. Kliment Ohridski”, 8 Dragan Tzankov Blvd., 1164 Sofia, Bulgaria; i.sazdova@biofac.uni-sofia.bg (I.S.); m_keremidarska@uni-sofia.bg (M.K.-M.); jsdanailov@uni-sofia.bg (Y.D.); 6 Department of Cell and Developmental Biology, Faculty of Biology, Sofia University “St. Kliment Ohridski”, 8 Dragan Tsankov Blvd., 1164 Sofia, Bulgaria; gn_georgiev@uni-sofia.bg (G.N.); r.konakchieva@biofac.uni-sofia.bg (R.K.)

**Keywords:** aging, NADPH oxidase, ROS signaling, anti-inflammatory, antiallergic, curcumin, irisin, melatonin, mitochondria, cyanotoxins

## Abstract

This review is focused on the mechanisms that regulate health, disease and aging redox status, the signal pathways that counteract oxidative and reductive stress, the role of food components and additives with antioxidant properties (curcumin, polyphenols, vitamins, carotenoids, flavonoids, etc.), and the role of the hormones irisin and melatonin in the redox homeostasis of animal and human cells. The correlations between the deviation from optimal redox conditions and inflammation, allergic, aging and autoimmune responses are discussed. Special attention is given to the vascular system, kidney, liver and brain oxidative stress processes. The role of hydrogen peroxide as an intracellular and paracrine signal molecule is also reviewed. The cyanotoxins β-*N*-methylamino-l-alanine (BMAA), cylindrospermopsin, microcystins and nodularins are introduced as potentially dangerous food and environment pro-oxidants.

## 1. Introduction

Optimal redox status is important for cellular functions and adaptation. Energy conversion and metabolic processes depend on cellular redox homeostasis. Oxidative and reductive stress are deviations from the optimal conditions when the capacity of cellular redox buffer systems is exceeded. Both conditions are harmful for cellular function and viability [1,2]. Different mechanisms are activated to restore the physiological values for the most common redox couples: NADH/NAD^+^, NADPH/NADP^+^ and reduced glutathione (GSH)/GSSG (oxidized glutathione), thioredoxin and for reactive oxygen species (ROS), such as superoxide anion (O_2_^•−^), hydrogen peroxide (H_2_O_2_) and hydroxyl radical (HO^•^), and reactive nitrogen species (RNS), such as nitric oxide (NO^•^) and peroxynitrite (ONOO^−^) derivative nitrogen dioxide [3]. Antioxidant systems include small molecules, such as ascorbate, α-tocopherol, GSH, many food additives and spices, the enzymes superoxide dismutases (SOD), catalase and glutathione peroxidases (GPx), proteins peroxiredoxins, thioredoxins and others [1,4,5,6].

This review is focused on the mechanisms of redox status in health and disease or aging, the signal pathways that counteract oxidative and reductive stress, the role of food components and additives with antioxidant properties (curcumin, polyphenols, vitamins, carotenoids, flavonoids, etc.), and the role of hormones melatonin and irisin in cellular redox homeostasis. The links between the optimal redox status deviation and inflammation, autoimmune responses, allergies and aging are discussed. Special attention is given to the involvement of oxidative stress processes in the function of the vascular system, kidney, liver and brain. The role of hydrogen peroxide (H_2_O_2_) as an intracellular and paracrine signal molecule is also considered. Several cyanotoxins are introduced as potentially dangerous food and environment pro-oxidants.

## 2. ROS and Blood Circulation

### 2.1. H_2_O_2_ as an Intracellular and Paracrine Mediator

ROS participate in the regulation of vascular function. The superoxide anion generates the more stable and membrane-permeable molecule H_2_O_2_, in a reaction that is catalyzed by SOD. H_2_O_2_ is a non-radical form of ROS with moderate oxidant activity that is a membrane-permeable paracrine regulator of vascular contraction [7,8,9,10]. It is produced in the endothelium, perivascular adipose tissue and smooth muscle cells [7,8]. H_2_O_2_ contracts rat coronary arteries by activating the Ca^2+^ influx in smooth muscle cells through L-type and non-L-type channels [7,10]. Additionally, it activates the regulatory pathways of Rho and MAP kinases and stimulates the production of the superoxide through NADPH oxidase (NOX) in the same tissue [7]. Similarly, in the rat skeletal muscle artery (*a. gracilis*), H_2_O_2_ changes the 5-hydroxytryptamine-induced contractions [8]. Our study, as well as others, observed the opposite effect of H_2_O_2_ on artery muscle tone, namely gradual vasoconstrictions in lower (physiological) concentrations and relaxation in higher concentrations [7,8]. The pleiotropic effects of H_2_O_2_ are linked to its ability to trigger the reversible oxidation of cysteine residues in proteins. Thus, it may change the activity of the key regulatory and functional molecules, such as the transcription factors, protein kinases and ion channels [11]. Therefore, H_2_O_2_ is considered to be an intracellular messenger.

### 2.2. ROS and Aging: NADPH Oxidase and Pathological Cerebrovascular Tone in Advanced Age

The brain circulatory system’s ability to maintain cerebral blood flow according to the variable metabolic demand to a greater extent relates to the contractile state of the cerebrovascular smooth muscle cells. As in other vascular beds, the contractile response of these cells is a function of the interaction between the actin and myosin filaments, catalyzed by phosphorylation of the 20-kDa regulatory light chain of myosin (MLC_20_) [12]. This reversible phosphorylation increases the ATPase activity of the actin–myosin complex, leading to so-called cross-bridge cycling, which is self-regulated by the activity of two specific enzymes: the myosin light chain kinase (MLCK), phosphorylating MLC_20_ in a Ca^2+^-dependent manner, and the myosin light chain phosphatase (MLCP), responsible for light chain dephosphorylation [13]. It is well-known that in vascular tissue the MLCP function can be altered via phosphorylation of its targeting subunit, MYPT1, leading to a change in the vascular tone, which is defined as a Ca^2+^-sensitization/desensitization [12,14,15]. Meanwhile, it is also appreciated that the contractile state of the vascular smooth muscle cells is regulated by actin polymerization, raising the actomyosin ATPase activity without change in the phosphorylation of MLC_20_ [13,16]. In addition, some actin-binding proteins, such as caldesmon, can directly inhibit actomyosin Mg ATPase activity and contraction at constant MLC_20_ phosphorylation perhaps via teetering of the actin and myosin myofilaments [17]. All these events are synchronized by modulating substances released from the vascular endothelium or perivascular nerves (or astrocytes depending on the vessel size), building a tree-component system that, together with the brain tissue, forms a functional domain, determined as a neurovascular unit [18]. In this functional unit, ROS produced by NOX orchestrate numerous cellular events, i.e., modifies the ion channels, kinases and phosphatases through post-translation, thus contributing to the physiological and pathological cerebrovascular tone regulation [19].

#### 2.2.1. Accelerated NOX Activity in the Regulation of the Contractile Activity of Cerebrovascular Smooth Muscle Cells

Numerous studies have proved that cerebral vessels show specific NOX expression patterns, with predominant expression of NOX1, NOX2, NOX4 and NOX5 in the smooth muscle cells [20,21,22,23]. While the role of NOX1, NOX2 and NOX4 in the development of cerebrovascular hypercontractility in animal models is well documented [23], recently it has been demonstrated that the major source of the ROS responsible for the oxidative stress and hypercontractility of human vascular smooth muscle cells relates to a specific NOX5 activation [24]. ROS accumulation is an important consequence of stroke and reperfusion, leading to inflammation and the immune response, which further damages neurovascular units [25]. Moreover, NOX-derived ROS and NO are involved in the development of angiotensin-II (ANGII)-evoked hypertension, well studied in animal models or human subjects [21,22,26].

Apart from the intensive studies, the exact underlying mechanisms of cerebrovascular smooth muscle cell contraction remain elusive. Biochemical investigations have shown that in human vascular smooth muscle cells, ROS modulate Na^+^ and Ca^2+^ conductance, as well as the activity of important kinases regulating the degree of MLC_20_ phosphorylation, such as focal adhesion kinase (FAK) and protein kinase C (PKC) [24,26]. It has been recently demonstrated that in advanced age, cerebral vessels undergo remodeling associated with inward hypertrophy and a rise in pressure-induced tone occurs [27]. This has been shown to be attributed to a rise in Ca^2+^-entry and the sensitivity of contractile filaments [28] and was prevented in a model of ANGII-induced hypertension by an NOX2 deficiency [22]. In this case, murine basilar arteries from old animals developed a hypercontractile phenotype, associated with a rise in the basal phosphorylation of MLC_20_, and counteracted by short-term treatment with the NOX inhibitor, apocynin [14]. Previous studies have demonstrated that vascular hypercontractility related to cerebral vasospasm or ROS accumulation implies the phosphorylation of the targeting subunit of MLCP, MYPT1, at the T696 and T853 phosphorylation sites, leading to MLCP inhibition and a rise in tone [29,30]. Importantly, apocynin inhibition of agonist-induced tone has blunted the translocation to the plasma membrane of the small G-protein RhoA, shown to be responsible for the activation of specific RhoA-dependent kinases (ROK) [31]. This observation has been supported by findings that in young and old basilar arteries the ROK sites of MYPT1 T696/853 are phosphorylated [14]. The same study corroborates the importance of ROK site T696, as the targeting of the threonine at position 696 into the non-phosphorylable alanine prevented the development of the hypercontractile phenotype in the basilar arteries of old animals [14]. Other studies have demonstrated NOX5-related hypercontractility after the direct phosphorylation of MLC_20_ via an activation by c-Src tyrosine protein kinase [24].

Recent studies using model systems have demonstrated that inhibiting the ROS/Src cascade reduces actin polymerization [24]. This is in line with the observation that old murine basilar artery polymerization of actin has also been accelerated [14]. In regard to another mechanism of the direct inactivation of the actomyosin complex via actin-binding proteins, it has also been demonstrated that NOX4-derived ROS reduces the expression of caldesmon in the primary smooth muscle cells and, thus, contributes to smooth muscle cell differentiation [32]. Others reported, when using an in vivo model, that ROS overproduction negatively regulates caldesmon expression [33]. Interestingly, in brain vascular tissue, caldesmon expression has strongly decreased as a long-term consequence of subarachnoid hemorrhage [34,35]. Moreover, it has also been demonstrated that the molecular targeting of caldesmon triggers cross-bridge cycling in unstimulated carotid arteries, a status that is typically associated with a hypercontractile response in the vasculature [36].

#### 2.2.2. NOX in Endothelial Dysfunction of Cerebral Vasculature

Several studies revealed that NOX activity directly affects the normal function of the cerebrovascular endothelium [23]. Compared to systemic arteries, here NOX-derived ROS has been shown to be an important relaxant, as NOX inhibition was able to prevent NADPH-induced relaxation and accelerate the contractile response of ANGII [20]. Meanwhile, it has also been reported that acute or chronic elevation of ANGII induces the development of a dysfunctional endothelial phenotype [21]. Authors have demonstrated that the underlying mechanism involves oxidative stress of the endothelial cells via NOX2 and, to some extent, NOX1 [19]. Regarding the fact that the ANGII level regulates aldosterone production, recently it has also been shown that systemic administration of the hormone increases endothelial ROS production via the activation of NOX2 [37]. Interestingly, here the degree of the ANGII effect on the superoxide production and blood pressure was significantly greater in aged mice, pointing out the role of aging in the development of endothelial dysfunction [37]. While the aforementioned studies focused mainly on ANGII-related pathology, others report a protective role of the sexual hormone estrogen against O_2_^•−^ production, supporting the existence of gender-related differences in NOX activity in cerebral circulation [38]. The protective role of estrogens has been additionally supported by the findings that exogenous estrogen administration reduces NOX activity by downregulating its regulatory subunits [38]. Interestingly, specific targeting of the ligand-inducible transcription factor peroxisome proliferator-activated receptor-γ (PPAR-γ) on the level of the cerebrovascular endothelium facilitates the detrimental effect of ANGII [39]. These findings support the notion that PPAR-γ plays the role of the endogenous endothelial protecting molecule, whose interference leads to endothelial dysfunction and hypertension [39,40,41].

Aging and ROS accumulation also cause major alterations to the third component of the neurovascular unit, the perivascular neurons [42]. Increased NOX activity has been shown to impede cerebral perfusion at macro- and microvascular levels and increase the risk of transient ischemic attacks, ischemic or hemorrhagic strokes, and dementia [42]. While the detrimental effect of ROS on cognition in relation to microcirculation is well documented [43,44], recently in animal models of cerebral artery occlusion and reperfusion, combating ROS accumulation using plant glycosides has also been shown to be able to restore cognition [45], pointing out the importance of brain macrocirculation for normal brain performance. In this context, histological studies have demonstrated that large cerebral arteries are innervated by preganglionic neurons originating from the sphenopalatine ganglion [46]. The latter excites small perivascular postganglionic neurons, releasing NO and a number of regulatory peptides [47]. In all species including humans, transmural electric stimulation or the application of nicotine lead to relaxation sensitive to hexamethonium, tetrodotoxin and nitric oxide synthase (NOS) inhibition, suggesting a nitrergic origin for this type of relaxation [46,47,48,49]. In this regard, in old basilar arteries, relaxation induced by nicotine was almost completely abolished and restored by short-term NOX inhibition using apocynin [14], demonstrating that the detrimental effect of ROS accumulation on the neurovascular unit is reversible.

Taken together, the presented data suggests that with advanced age, ROS accumulation in the smooth muscle cells accelerates cerebrovascular contractility via the phosphorylation of MLC_20_ in the thick myosin filaments, as the underlying mechanisms of smooth muscle hypercontractility involve the activation of the signal cascades, ROS/c-Src or ROS/RhoA/ROK/MYPT1. Studies on actin polymerization or protein caldesmon corroborate the notion that hypercontractility associated with the thin contractile filaments occurs and may, additionally, contribute to pathological cerebrovascular tone activation in advanced age. Moreover, combating pathologic ROS accumulation using estrogen or restoration of the activity of the endogenous PPAR-γ or NOX inhibition alleviates the effects of cerebrovascular endothelial dysfunction and restores neurovascular coupling in the vessels of aged animals. All these findings provide a strong rationale for future therapeutic strategies involving the treatment of aging-related cerebrovascular disease based on NOX targeting.

#### 2.2.3. ROS and Vascular Remodeling: The Role of Sestrins, Uncoupling Protein 2 and PDGF

Oxidative stress is a major contributor to the development of cardiovascular diseases, such as atherosclerosis and hypertension [50]. The Sestrin family of proteins plays a crucial role in regulating cellular metabolism and the stress response, protecting cells from oxidative stress-induced damage and reducing inflammation [51]. Sestrins have been shown to promote vasodilation and reduce vascular remodeling, suggesting a potential role for them in preventing cardiovascular diseases [51,52]. Oxidative stress can impair Sestrin expression and function, which may contribute to the development of vascular diseases [52,53]. Thus, maintaining Sestrin expression and function may be a potential therapeutic target for preventing or treating cardiovascular diseases related to oxidative stress.

Uncoupling protein 2 (UCP2) regulates cellular metabolism and energy balance, particularly in the context of oxidative stress [54]. UCP2 can protect cells from oxidative stress-induced damage by regulating the mitochondrial function and reducing the production of ROS [55]. However, under chronic oxidative stress conditions, UCP2 can contribute to vascular remodeling by promoting vascular smooth muscle cell (VSMC) proliferation and migration [56,57]. This may lead to the development of vascular diseases, such as atherosclerosis and hypertension [58]. Therefore, the role of UCP2 in vascular remodeling is complex and context dependent, and its modulation may be a potential therapeutic strategy for preventing or treating cardiovascular diseases related to oxidative stress.

The platelet-derived growth factor (PDGF) is a potent mitogen and chemoattractant for VSMCs and plays a critical role in vascular remodeling in response to injury or chronic oxidative stress [59,60]. Oxidative stress can stimulate PDGF signaling by increasing the production of ROS, leading to increased VSMC proliferation and migration and, ultimately, contributing to the development of vascular diseases, such as atherosclerosis and restenosis [58,61]. PDGF signaling also contributes to the activation of pro-inflammatory pathways and the formation of atherosclerotic plaques [62,63]. Therefore, targeting PDGF signaling may be a potential therapeutic strategy for preventing or treating cardiovascular diseases related to oxidative stress.

Oxidative stress can induce vascular remodeling through different mechanisms related to Sestrins, UCP2 and PDGF. Targeting these signal pathways may be a potential therapeutic strategy for preventing or treating cardiovascular diseases related to oxidative stress-induced vascular remodeling.

### 2.3. ROS and Renal Vascular Tone Regulation

The role of ROS in the renal vascular function has been recently extensively reviewed, with particular emphasis on the afferent arteriolar function [64]. In the vasculature, NOX is the major source of ROS and this is also true for the renal blood vessels [64,65]. In afferent arterioles, NOX2-dependent superoxide formation contributes to the development of vasoconstriction elicited by angiotensin II [66]. Also, the endothelin-1-induced vasoconstriction in the renal microcirculation depends on superoxide, whose source is not yet determined [67,68]. Furthermore, sphingosine 1-phosphate-induced vasoconstrictions of afferent arterioles could be inhibited by the NOX inhibitor apocynin, suggesting that NOX-dependent superoxide formation contributes to this vasoconstriction [69]. In the descending *vasa recta*, the inhibition of NOS induces vasoconstriction that could be offset by the SOD mimetic tempol or apocynin. In this regard, a word of caution is needed regarding the data on vascular tone regulation obtained with apocynin, since it requires high peroxidase activities to inhibit NOX, which may not always be present in vascular preparations [70]. Apocynin also has an antioxidant activity [71] and inhibits eNOS-dependent superoxide generation. Additionally, several other effects of apocynin are reported in the diabetic and cancer model system. They include reduced activity of NOD-, LRR- and pyrin domain-containing protein 3 (NLRP3), P38/MAPK/Caspase3, NF-κB, as well as reduced signaling by transforming growth factor beta (TGFβ), AKT-GSK3β, ERK1/2 and PI3K/Akt signaling [72]. These pleiotropic influences of apocynin require more cautious interpretation of the data obtained using this acetophenone.

In more proximal parts of the renal vasculature, such as the rat renal interlobar arteries, high expression of NOX2 and NOX4 has been shown in the endothelium [73]. Moreover, NOX2 and NOX4-dependent H_2_O_2_ formation has been demonstrated to contribute to endothelium-dependent vasodilation in rat interlobar arteries [73]. Data on vascular NOX and the contribution of ROS to vascular tone regulation in the human kidney are sparse. NOX2 and NOX4 mRNA are expressed in human arcuate and proximal interlobular arteries [65], while immune-histochemical studies demonstrated NOX5 in small human intrarenal arteries obtained from biopsies [74]. Administration of the superoxide scavenger tiron did not affect the phenylephrine and endothelin-1-induced vasoconstrictions in human arcuate and interlobular artery segments, while it reduced endothelium-dependent vasodilation in these vessels [65]. The latter findings were corroborated by a more recent study showing that apocynin blunted the endothelium-dependent vasodilation in human interlobar arteries [73]. Together, these findings suggest that ROS, probably H_2_O_2_ [73], contribute to endothelium-dependent vasodilation in human intrarenal arteries.

## 3. Antioxidants in Diabetes, Vascular Injury, Hypoxia, Atherosclerosis and Allergies

### 3.1. Enzymatic and Non-Enzymatic Antioxidants

Oxidative stress and its consequences are mitigated by antioxidants, either as part of the body’s natural defense mechanism or obtained from various dietary sources. Antioxidants can generally be categorized into two main groups: enzymatic and non-enzymatic. Enzymatic antioxidants, such as SOD, which catalyze the dismutation of O_2_^•−^ into H_2_O_2_ and O_2_, catalase, which catalyzes the H_2_O_2_ hydrolyzation into H_2_O and O_2_, GPx, which catalyzes the hydrolyzation of H_2_O_2_ into H_2_O and O_2_ and the reduction of ROO• into alcohols and O_2_, or glutathione reductase, which catalyzes the reduction of GSSG to GSH, provide a mechanism of eliminating ROS, thus preventing cellular damage. Hydrogen peroxide, alkyl hydroperoxides and peroxynitrite are reduced by peroxiredoxins [75]. They are recycled by thioredoxin, which reduces the oxidized cysteine residues and is itself recycled by thioredoxin reductases. There are multiple other non-enzymatic substances with antioxidant activities, such as vitamins, namely vitamin C (ascorbic acid), vitamin E (tocopherol), and vitamin A (retinol), cofactors, namely vitamins B1, B2, B6, B12, folic acid, glutathione, minerals, namely copper, zinc, manganese and selenium, and various other compounds (carotenoids, flavonoids) absorbed from plant-based nutritional sources [76,77].

Studies have shown that natural antioxidants have the potential to improve endothelial dysfunction and reduce inflammation, which are vital contributors to the vascular complications associated with diabetes. Some antioxidants can directly scavenge ROS or modulate the signaling pathways involved in the regulation of oxidative stress. Others activate the nuclear factor erythroid 2-related factor 2 (Nrf2) signaling pathway, which upregulates antioxidant and cytoprotective gene expression. Antioxidants can also modulate the activity of the enzymes involved in the production of vasoactive compounds, such as NOS and cyclooxygenase (COX) [78,79,80,81].

Vitamins A, C and E are three essential antioxidants that scavenge free radicals and enhance the activity of other antioxidants, thus preventing the propagation of lipid peroxidation and membrane damage [82,83]. A meta-analysis by Ashor et al. [84] demonstrated that supplementation with these vitamins significantly reduces arterial stiffness, which can be explained by the reduction of the effect of free radicals on the components of the vessel walls [85].

Plants are rich in various polyphenols with antioxidant properties that have beneficial effects on DM-associated vascular complications. For example, resveratrol, found in grapes and berries has been shown to reduce inflammation and improve insulin sensitivity and vascular dysfunction in both animal and human studies [86,87,88]. It acts as a potent antioxidant that inhibits the nuclear transcription factor kappa B (NF-κB) signaling pathway concomitantly with suppressing the expression of hypoxia-inducible factor 1 alpha (HIF-1α) and the vascular endothelial growth factor (VEGF), thus having pleiotropic effects on a variety of medical conditions related to inflammation, metabolic imbalance or cancer [89,90].

**Figure 1:**
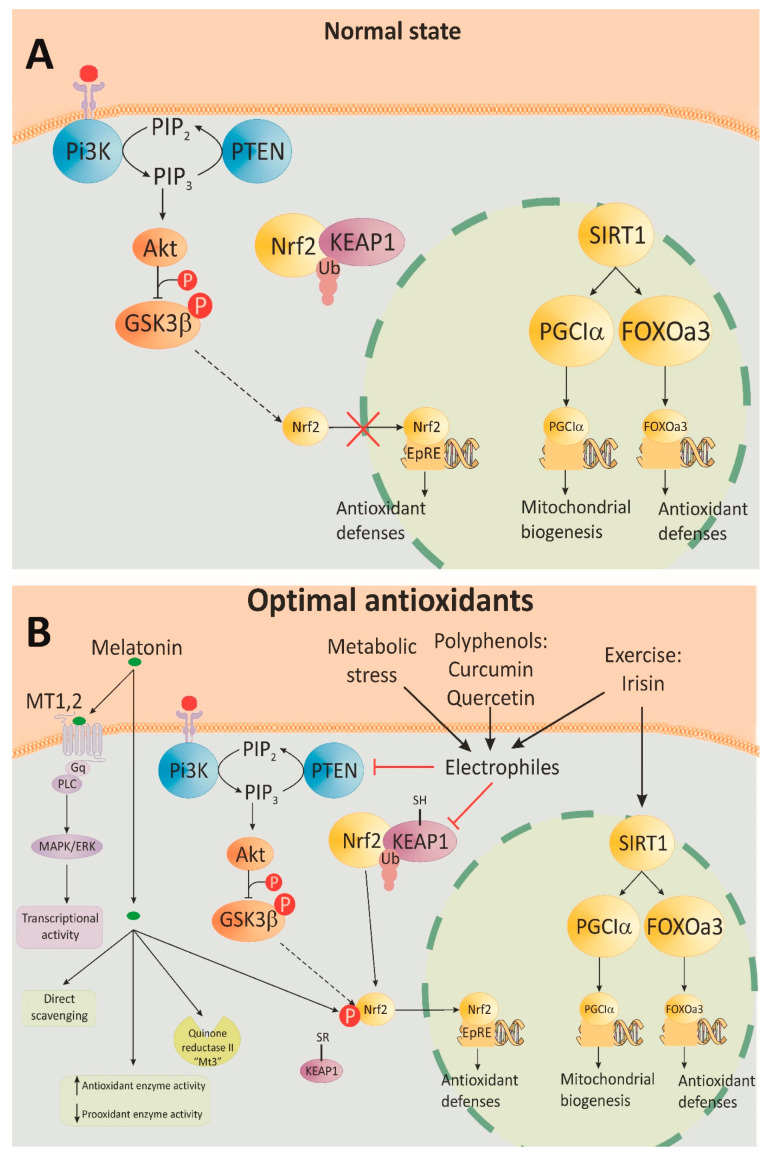
Nrf2 and SIRT1 signaling pathways regulation by antioxidants. (**A**) At optimal conditions, Nrf2 forms a KEAP1-ubiquitin complex, which downregulates Nrf2 via proteasomal degradation. Nrf2 activity is also inhibited by PTEN. This phosphatase decreases 3-phosphoinositides (PIP3) by conversion into PIP2, and the downstream Akt-GSK3β are not activated. The small amount of Nrf2 cannot be phosphorylated and translocated into the nucleus. Another mechanism of antioxidants involves SIRT1/FOXOa3 and the PGC1α pathway by expression of the antioxidant defense genes and mitochondrial biogenesis. (**B**) With physiological doses of antioxidants, KEAP1 is oxidized and the Nrf2 complex is destroyed, which results in an increased amount of Nrf2. PTEN is also suppressed and, thus, activates the previously described pathway with the following translocation of Nrf2 into the nucleus. Phosphorylated Nrf2 binds to the electrophile response element (EpRE) that starts the expression of the phase II antioxidant enzymes. Metabolic stress activates the Nrf2 pathway in the same manner. Moderate physical exercise stimulates both Nrf2 and SIRT1 signaling. Melatonin exerts its antioxidant effects through receptor-mediated transcriptional activity and after diffusion in the cytosol via several different pathways.

Multiple studies on the effect of curcumin, a natural polyphenolic compound derived from *Curcuma longa*, demonstrate pluripotent effects on oxidative stress, insulin sensitivity and cardiovascular health in people with diabetes. The mechanism of action of curcumin involves suppression of the p300/CREB-binding protein and PKC expression and modulation of multiple signaling pathways, including MAPK, JAK2/STAT3, c-Jun/AP-1, Nrf2 and Src/Akt, among others [91,92,93], (Figure 1). Epigallocatechin 3-gallate (EGCG), found in green tea as well as in cranberries, strawberries, blackberries, kiwis and other fruits, has insulin-mimetic actions on glucose metabolism and improves oxidative status. It has shown beneficial effects on vascular complications from diabetes, such as retinopathy, nephropathy and cardiovascular disease [94,95,96,97,98,99]. EGCG improves mitochondrial dysfunction, inhibits the formation of ROS and acts as a free radical scavenger [100,101,102]. The mechanism of action of EGCG includes pleiotropic activation of the phosphoinositol 3-kinase (PI3K), Akt, AMPK and eNOS signaling pathways and stimulation of the endothelial production of NO [103,104]. Human studies support the beneficial effects of EGCG on cardiovascular health. Acute supplementation with EGCG reversed endothelial dysfunction in patients with coronary artery disease [105], and supplementation of early atherosclerosis patients with olive oil rich in EGCG and other plant-derived polyphenols improved endothelial function [106].

### 3.2. Diabetes Mellitus

Diabetes mellitus (DM) is a chronic metabolic disorder characterized by high glucose levels resulting from insulin deficiency or insulin insensitivity. According to the International Diabetes Federation [107], today, diabetes affects more than 500 million people worldwide. One of the main consequences of chronic hyperglycemia is vascular dysfunction, characterized by a variety of abnormalities in the structure or function of the blood vessels. These complications include microvascular damage affecting the retina and the kidneys and macrovascular changes leading to atherosclerosis, coronary artery disease and stroke. Epidemiological data show that DM increases cardiovascular disease risk up to 8-fold [108].

It is widely accepted that DM-induced vascular dysfunction is caused, among others, by oxidative stress. Oxidative stress arises as a result of an imbalance between the generation of ROS and the capacity of the cells to detoxify and repair damage to cellular proteins, lipids and DNA, which results in cellular malfunction and death. Practically every aspect of DM (hyperglycemia, inflammation, dyslipidemia and mitochondrial dysfunction) can cause oxidative stress that can exacerbate the diabetic state, leading to vascular complications.

Even though the link between diabetes and oxidative stress is complicated and has many parts, there are several ways that diabetes can damage blood vessels [109]. On the one hand, hyperglycemic conditions in DM can cause an increase in the polyol pathway flux that can lead to the formation of ROS and cause a decrease in NO availability. Aldose reductase is part of the polyol pathway, which converts glucose into sorbitol. Disruption of this pathway leads to a buildup of sorbitol in the cells, resulting in oxidative stress, inflammation and increased adhesion of monocytes and macrophages to the endothelial cells [108]. Persistent hyperglycemia also causes activation of the NF-κB signaling pathway and an increase in NO production, which react with the superoxide anion radicals to form reactive peroxynitrites [82]. On the other hand, high glucose levels activate PKC isoforms and proinflammatory cytokine and prostaglandin expression, thus causing oxidative stress, inflammation, endothelial dysfunction, plaque formation and atherosclerosis. Additionally, hyperglycemia leads to protein and lipid modulation and the formation of advanced glycation end-products, concomitantly with the activation of the hexosamine pathway flux, which can further intensify ROS production and inflammation [109,110,111]. Dyslipidemia, defined by elevated levels of circulating triglycerides and low-density lipoprotein (LDL) cholesterol, induces oxidative stress via the activation of NOX [112]. Moreover, mitochondrial dysfunction in DM inhibits electron transport chain activity and enhances the uncoupling of oxidative phosphorylation, which can further upregulate ROS generation.

### 3.3. Antiallergic Potential of Curcumin and Tetrahydrocurcumin: Structural Features, Signaling and Supplementary Properties

Orally taken curcumin (CUR) is converted into tetrahydrocurcumin (THC) or its conjugated forms [113,114]. Different animal studies have shown that CUR possesses anti-inflammatory and antiallergic properties [115]. These effects are usually associated with suppression of the production of prostaglandins (PGs), leukotrienes (LTs) [115,116], NO, [117] and cytokines (IL-16, IL-5, and TNF-α), as well as the inhibition of histamine release from mast cells [118,119].

Suzuki et al. (2005) hypothesized that numerous pharmacological actions of CUR are based on its antioxidant properties [120]. Thus, by using an in vitro approach, they have determined the antiallergic and antioxidant activities of various CUR derivative compounds and further investigated the relationships between these two activities. Specifically, they studied CUR, THC and some of their glycosides, showing the inhibition of histamine release from a commonly used histamine-releasing cell line (RBL-2H3) induced by concanavalin A or calcimycin (A23187). The obtained results confirmed that various CUR analogs can act in the process of degranulation, after the entry of Ca^2+^ into the mast cells, thereby causing the inhibition of the histamine release [120]. Moreover, the same authors showed that CUR inhibited histamine release with the same intensity as THC. In the literature, however, CUR was more potent than THC in inhibiting PGE2 generation or NO production [121,122,123]. Conversely, THC was more potent than CUR in the induction of antioxidant responses [113,124]. It is also well-known that ROS are necessary for the induction of inflammation. Macrophage-generated ROS can induce the production of PGE2, NO and cytokines (IL-1α, IL-6, and TNF-α), which initiate inflammation [125,126,127]. TNF-α induces ROS production, which triggers inflammatory conditions and endothelial disfunction, and can change VSMC from the contractile into the secretory phenotype [128]. Further, the free radicals released from the metabolites of unsaturated fatty acids also induced histamine release in rat mast cells [129,130,131]. CUR treatment has been shown to inhibit ROS release from macrophages [132] and reduce histamine release from mast cells [7,8]. These findings indicate that the antiallergic properties of CUR are closely related to its free radical scavenging properties. One of the most important non-antioxidant dependent mechanisms related to the antiallergic effects of CUR is the inhibition of PKC, phospholipase A2 and phospholipase C, [116,133,134], as well as COX and 5-lipoxygenase (5-LO) [115,135,136].

#### 3.3.1. Structurally Associated Antiallergic Properties of CUR and THC

**Figure 2:**
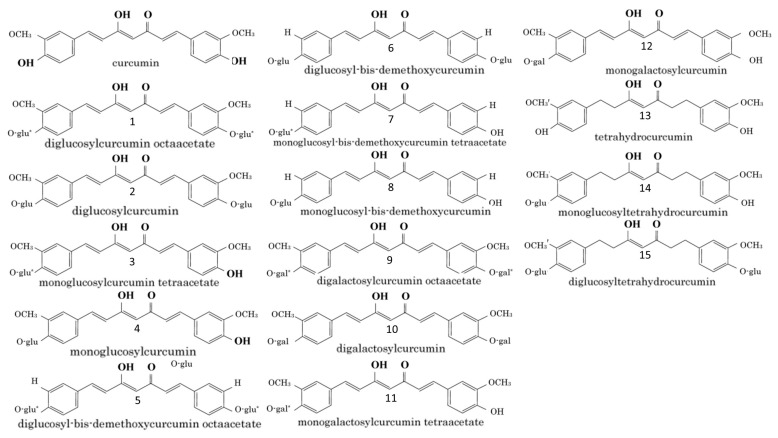
Chemical structures of curcumin related compounds. Abbreviations: glu: glucose; gal: galactose; *: tetraacetate or octaacetate.

Recent data clearly show that when administered orally, CUR retains its antiallergic activity, despite its extensive metabolism of THC. Structurally, it contains two methoxy groups, two phenolic hydroxy groups and three conjugated double bonds (Figure 2). Current studies have shown that the potency to inhibit the release of histamine does not depend on the reduction of the number of conjugated double bonds, but rather on the high potency of THC. On the other hand, Futagami et al., (1996) report equivalent inhibitory activity of dimethoxy-CUR and CUR to histamine release [119]. However, phenolic glycoside analogs of CUR and THC show weaker potency in inhibiting histamine release, while their tetraacetate or octaacetate derivatives have a negligible inhibitory effect on histamine release [121]. Based on this, it has been established that the phenolic hydroxy groups of CUR and THC play a key role in inhibiting histamine release. The antioxidant activity of CUR and its monoglycoside is significant, but it is not so for the diglycoside and bis-dimethoxy analog of CUR [120]. THC shows similar results. Such results highlight the important role of phenolic hydroxy and methoxy analogs of CUR in the development of antioxidant capacity [120,137,138]. This is related to the fact that the antiallergic activity of CUR is in part due to its antioxidant activity. However, it should be noted that some antioxidant-impotent analogs [102,114,120,138] show distinct antiallergic activity. On the other hand, the compounds possessing tetraacetate in their chemical structures (Figure 2) [115,123], are not characterized by inhibitory effects on the release of histamine, despite their ability to prevent the production of free radicals [120,137,138]. Compounds 3 and 11, compared to compounds 4 and 12, whose structures lack tetraacetate, have higher molecular weight and lower solubility in water. This likely affects their passage through the membrane, resulting in very low antiallergic effects in cells.

#### 3.3.2. THC Associated STAT6-Dependent and STAT6-Independent Signaling in Airway Allergic Reactions

Allergic asthma is a chronic inflammatory disease broadly defined by increased inflammatory infiltrates, mucus production, bronchoconstriction and airway hyperreactivity [139]. A pronounced Th2 response, as a consequence of eosinophilic tissue infiltration, is the primary inflammatory phenotype in allergic asthma. The first line of protection mainly includes the use of bronchodilators and inhaled or oral corticosteroids, but their use is limited due to numerous side effects [138]. It seems that there have been great advances in the treatment of asthma (such as the anti-IL-5/IL-13 antibody) [139,140,141], but antibody-based treatments also include certain limitations, as: (1) the identification of potentially responsive patients based on these biomarkers is required, (2) adverse effects may occur even after discontinuation of therapy, and (3) treatment with monoclonal antibodies is still very expensive. Hence, the need to develop new drugs and strategies to treat asthma is enormous. We, and others, have shown that different CUR analogs can modulate airway disorders, such as bronchopulmonary dysplasia, chronic obstructive pulmonary disease, asthma and pulmonary fibrosis, through the induction of multiple mechanisms, which are related to its anti-inflammatory, antioxidant and antibacterial properties [142,143,144,145,146]. The approach in the synthesis of new structural analogs of CUR proved to be one of the most elegant in improving the low bioavailability of CUR [147].

**Figure 3:**
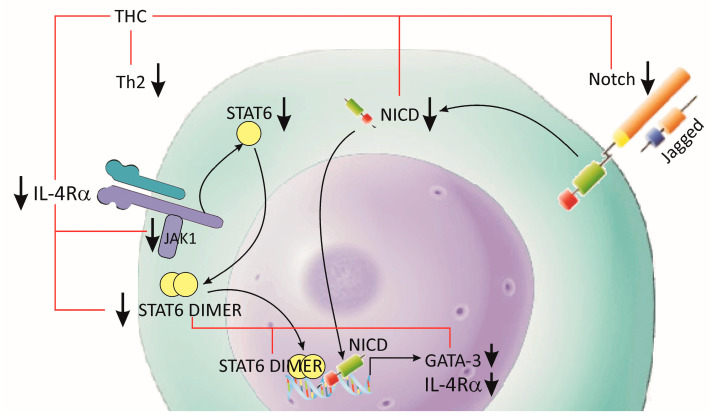
Effects of THC on Th2 differentiation. Allergic inflammation and asthma are characterized by pathological Th2 cell activation. THC treatment reduced the expression of IL-4Rα and GATA3, and the phosphorylation of Jak1 and STAT6 in Th2. The inhibitory effect of THC was additionally supported by the downregulation of Jagged1/Jagged2 and NICD1/NICD2 signaling.

CD4+ T cells are critical components of the adaptive immune response. They play an important role in the recruitment and activation of other immune cells, dampening the current immunological responses and the maintenance of immunologic memory. Following activation of the T cell receptors and co-stimulation by antigen-presenting cells (APC), I CD4+ T cells develop into one of multiple T helper cell subtype lineages. These subgroups have distinct transcription factors, cell surface proteins and secreted molecule combinations. T helper type 2 (Th2) cells defend the host against intestinal helminths and external microorganisms, while also supporting B cell-dependent humoral responses. Recent studies showed that the signal transducer and activator of transcription 6 (STAT6)-dependent IL-4/IL-4Rα/Jak1-STAT6 and STAT6-independent Jagged1/Jagged2-Notch1/Notch2 signaling pathways play a key role in the inflammatory processes during allergic reactions in the airways [148,149], (Figure 3). A study by Chen et al. (2018), showed that THC treatment reduced the expression of the interleukin receptor 4α (IL-4Rα) and transcription factor GATA3, and the phosphorylation of Janus kinase 1 (Jak1) and STAT6 in local leukocytes [148]. The effect of THC was more pronounced in the inhibition of the STAT6-independent pathway, Jagged1, Jagged2 and the activated forms of Notch 1 (NICD1) and Notch 2 (NICD2) [148]. The same group of authors is of the opinion that the reduction of Th2 cells after THC treatment is partially caused by the inhibition of GATA3 through the STAT6-dependent and STAT6-independent signaling pathways. Interestingly, they showed that treatment with THC or CUR suppressed the Th17 and Tc17 cell subsets, but did not modulate either the Th1 or regulatory T-cell (Treg) responses [148]. It should be taken into account that Th17 cells have high plasticity and flexibility during inflammatory processes, i.e., they differentiate and induce transition into other T-cell phenotypes through cytokine expression (such as intermediate Th1/Th17 cells and Tregs) [149,150,151,152].

The studies presented above confirmed for the first time that the bioavailability of THC is significantly higher in mice treated with THC than in those treated with CUR. It has been unequivocally shown that THC is more effective than CUR in alleviating the symptoms associated with the allergic reaction. THC has also been shown to inhibit the growth of Th2 cell lines, as well as the production of Th2 cytokines IL-4 and IL-5. The inhibitory effects of THC on IL-5 levels have a key role in the prevention of eosinophilic infiltration [153]. Numerous studies have also shown more pronounced antioxidant effects of THC in comparison with CUR [144,145,146]. Taken together, these results are all in favor of the fact that THC is far superior to CUR in the treatment of allergic asthma.

#### 3.3.3. THC Potentiation of the Therapeutic Effects of Corticosteroids in a Mouse Model of Allergic Asthma

In their study involving asthmatic mice, Wu et al. (2020) showed that THC supplementation has similar effects to treatment with the corticosteroid dexason (DEX) in the regulation of the inflammatory processes of the airway, as well as in the regulation of the pathological changes in the lung [154]. In addition, the same group found that THC enhanced the therapeutic effects of DEX compared to monotherapy (THC alone or DEX alone), manifested, among others, by lower mucus production and a weaker Th2 and Th17 response. Hence, it appears that supplementation with THC may have a potential application in clinical use.

Besides the numerous limitations to glucocorticoid therapy, minimizing potential side effects involves various approaches, with the safest being demonstrated in the combination therapy of DEX with nutrients, which (1) enhances the therapeutic effects of DEX, (2) reduces the required dosage of DEX, and (3) prevents or at least alleviates the side effects caused by DEX.

The obtained results from recent studies indicate that THC is better tolerated compared to DEX, which is probably due to the good response in humans to CUR even at high doses of up to 12 g/day [155], while toxicity studies in rats showed no harmful effects of THC up to 400 mg/kg/day. Thus, THC can be considered an alternative therapy for allergic asthma.

Shen et al. (2018) revealed the immuno-modulatory properties of THC against asthma depended on the inhibition of the Th2 response due to the downregulation of the IL-4Rα-Jak1-STAT6 and Jagged1/Jagged2–Notch1/Notch2 pathways [155]. Considering that DEX treatment can induce side effects [156,157], such as deterioration of the articular cartilage [158], the alleviation of vascular dysfunction and blood pressure [159], these findings suggest a potential protective role of THC against DEX-induced side effects, and that a combination therapy involving THC and DEX may be safer than DEX alone in the treatment of allergic asthma. Whether a higher dose of THC can further enhance the therapeutic effects of DEX and reduce the side effects remains unknown, and more pharmacological experiments should be conducted in the next phase.

In general, the hydroxyl groups of CUR may play a significant role in exerting both antioxidant and antiallergic activities. At the same time, some of the CUR analogs do not possess antiallergic activity despite their antioxidant characteristics.

THC exhibits a more dominant anti-inflammatory efficacy than CUR, which qualifies it as a bioactive product that overcomes the limitations of CUR. Oral administration of THC alleviates airway inflammation by reducing symptoms like eosinophilic infiltration, the generation of Th2-associated cytokines and Th2 and Th17 responses, as well as the suppression of the Th2 signaling pathways IL-4/L-4Rα/Jak1-STAT6 and Jagged1/Jagged2–Notch 1/Notch2.

Different pharmacological activities of the various CUR analogs are now under investigation in further studies, where it is expected that light will be shed on the antiallergic mechanisms. Thus, the therapeutic benefits of THC in the treatment of allergic airway reactions are promising and will fuel further studies to determine its clinical significance. In this direction, combined therapy with THC and DEX showed superior therapeutic implications, such as higher therapeutic effects than monotherapy and reduced use of glucocorticoids to avoid the related side effects.

### 3.4. Flavonoids

Flavonoids are a group of natural phytophenolic compounds, based on 15 carbon skeletons consisting of two benzene rings connected via a heterocyclic pyran ring. There are variations in the level of oxidation and the substitutions of the benzene rings classified into the following subgroups: flavones, flavonols, flavanonols, flavanones, isoflavones, flavan-3-ols, anthocyanidins, chalcones and aurones [160]. Natural sources of flavonoids are multiple plant seeds, stems, leaves, fruits and flowers, where these compounds are responsible for the attraction of pollinators, and protect from UV rays, freezing and pathogens [161]. Besides the well-known functions of flavonoids in plants, it turns out that these compounds play an important role in inflammation, allergies and oxidative stress in animals and humans. They are able to inhibit the mitogen-activated protein kinases (MAPKs) and NF-kB, the key modulators in the expression of several pro-inflammatory genes [162]. Some flavonoids suppress the activity of Th2 cells via the transcription factors GATA-3 and STAT-6 [163]. Flavonoids from ginger, ginkgo biloba and artichoke inhibit phosphodiesterases, which is another mechanism influencing inflammation and chronic allergies [164]. Sudachitin is a specific flavonoid in some citruses that has been shown to suppress lipopolysaccharide-induced inflammation in mouse macrophage-like RAW264 cells and decrease the levels of TNF-α and nitrates produced in these cells [165]. Lots of research data present the direct effects of flavonoids on different immune cells by hindering their proliferative and adhesive properties, and by reducing histamine, prostaglandins and cytokines secretion and the production of IgE antibodies [166]. In addition, they act as potent scavengers of free radicals generated during the inflammation process [167,168,169].

Quercetin, a flavonoid found in various fruits, vegetables and grains, has been shown to normalize glucose levels, inhibit inflammation and oxidative stress, and improve endothelial function. Quercetin suppresses PKC, blocks calcium channels and modulates cytokine expression [87,170]. Silymarin is an extract from the plant *Silybum marianum*, which contains a combination of flavonolignans, such as silidianin, silibinin, silicristin and isosilibinin. It is used for renoprotection against oxidative stress and inflammation in different in vitro and in vivo animal and human models of chronic kidney disease and diabetic nephropathy [171]. Luteolin, a flavonoid that is abundant in carrots, onion, celery, apples and chamomile, demonstrated hepatoprotective activity in Pb-intoxicated rats due to the upregulation of Nrf2 expression and the murine double minute 2 (Mdm2) gene, together with the suppression of p53 expression [172].

The antioxidant and anti-inflammatory properties of flavonoids make them valuable dietary compounds in the prevention of cardiovascular diseases. Flavonoids act as antagonists of thromboxane A2 receptors, protect the collagen in blood vessels against oxidative stress, block the rise of intracellular calcium and prevent platelet aggregation through multiple pathways [173]. Many flavonoids induce vasodilatation through stimulation of NO production and, thus, increase the plasma NO level. Hesperetin can activate the voltage-gated ion channels in the vascular smooth muscles and, thus, have an antihypertensive effect through a hyperpolarization-dependent decrease in the calcium influx [174].

Many other naturally derived compounds with antioxidative properties have been utilized in traditional medicine or for culinary purposes for centuries. These compounds have been extensively studied, chemically modified and formulated as clinically approved medications. It is estimated that approximately half of the currently used medications originate from plants [175]. One notable example is metformin, one of the most prescribed drugs for treating type 2 DM (T2DM), which is synthesized through a chemical modification of guanidine found in *Galega officina*. Another class of medications inspired by nature is the group of thiazolidinediones.

### 3.5. Overdoses of Polyphenols

Supplements containing polyphenols have antioxidant or pro-oxidant properties, depending on the dose level and the biological environment [176,177,178]. At normal physiological conditions, the thioredoxin and glutathione systems are the first line of defense against excessive polyphenol-induced stress. Toxic levels of polyphenols strongly activate the Nrf2 system in selenium-deficient conditions. The side effects of excessive polyphenol consumption are a result of their auto-oxidation and ROS (H_2_O_2_ and superoxide anion) production. Furthermore, copper and iron ions promote the oxidation of polyphenols and, thus, amplify the production of ROS [179,180]. They are also transformed into quinones and semiquinones, which covalently bound to the free thiol group of cysteine residues in proteins, leading to the formation of quinoproteins [181]. Toxic and lethal doses can be reached when consumed in isolated form as dietary supplements rather than as plant food. These negative effects of antioxidants have been assigned to the term “antioxidative stress” [182]. Vitamins C and E, SOD, GSH and beta-carotene have a potentially harmful effect due to antioxidative stress. The most common ROS and NO are signaling molecules, which regulate transcription factors and the activity of any SH-containing molecule (GSH, PKC, Ca^2+^-ATPase, collagenase and tyrosine kinases). For this reason, the complete elimination of ROS compromises normal cell signaling and function [183].

**Figure 4:**
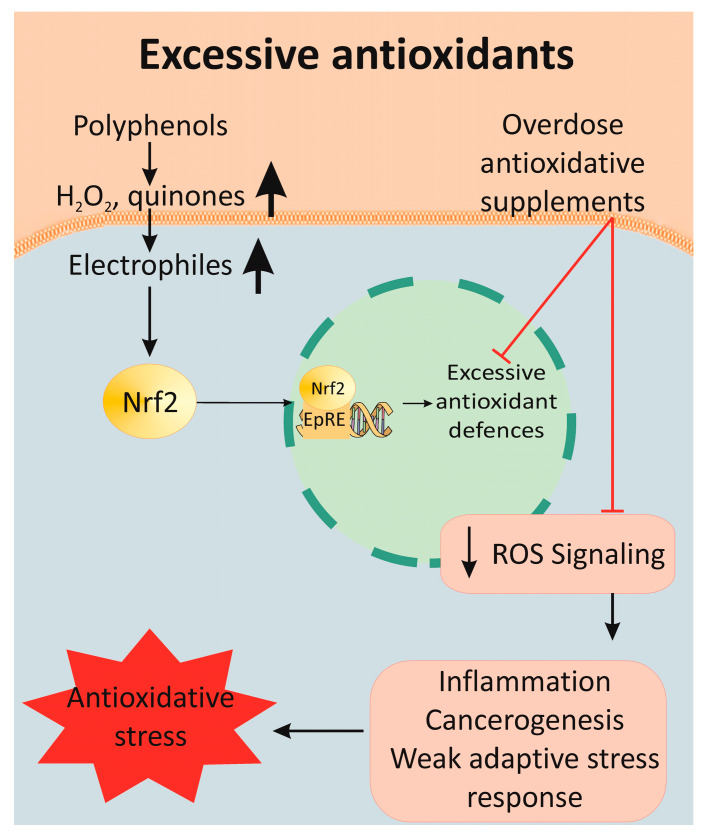
Mechanism of antioxidative stress after supplementation with polyphenolic overdoses. Many antioxidants lead to the overexpression of antioxidant enzymes, which dramatically decreases the ROS signaling pathway. As a result, the induction of an inflammatory response, carcinogenesis and instability in the cell redox balance are observed, all of which are collectively determined as antioxidative stress.

Antioxidants activate numerous enzymes for antioxidative defense through SIRT2/FOXO, NF-κB and Nrf2/ARE signal pathways (Figure 4). The same mechanisms are activated by moderate, intermittent stress factors, such as exercise and energy restrictions. Much data confirm the relationship between energy restriction and stimulation of the antioxidant defense, which increases peroxidative stress resistance [184]. Moderate physical exercise enhances mitochondrial activity and ROS production. The latter initiates an adaptive stress response, which results in improved health. On the other hand, simultaneous antioxidant supplementation and physical activity can reduce its positive effect on health [185].

## 4. Irisin: More Physical Exercise for a Longer and Better Life

### 4.1. Irisin as a Myokine Hormone

Irisin is a cleaved product from the fibronectin type III domain-containing protein 5 (FNDC5) that is composed of 112 amino acids and is glycosylated. This is a myokine hormone released from skeletal muscles after intensive exercise [186]. It has been suggested that irisin protects against obesity and insulin resistance due to its ability to stimulate energy consumption by increasing the number of mitochondria and the expression of the uncoupling protein-1 (UCP-1) in white adipocytes that converts them into a brown adipocyte-like phenotype [186]. As a result, glucose homeostasis is improved, which leads to an anti-obesity effect [187]. Additionally, the decrease in visceral adipose tissue reduces a wide variety of adipose tissue-derived hormones (mainly adipokines) that promote hypertension, inflammation, oxidative stress and cellular aging [188,189] and the references therein]. The hypothesis for a direct anti-aging effect of irisin is supported by the strong correlation between the relative telomere length, a genetic marker of aging, and the plasma level of irisin in healthy adults [190]. In humans, the conversion of white into brown adipocytes in abdominal subcutaneous fat tissue is much less available when compared to rodents, and the expression of UCP-1 is weak [191].

### 4.2. Irisin as an Antioxidant, Anti-Inflammatory, Anti-Atherosclerotic and Anti-Aging Mediator

The number of beneficial effects and target tissues of irisin has been increasing [192] since its identification in 2012 [186]. In mice, irisin enhances bone formation by osteoblasts and decreases bone resorption by osteoclasts [193]. Reduced expression of the FNDC5 mRNA and irisin caused by mechanical unloading leads to bone loss. Lower levels of irisin removed its inhibitory effect on the formation of osteoclast from mouse bone marrow cells, leading to bone resorption [194]. The positive link in the musculoskeletal system (irisin-stimulated bone formation) is indirectly supported by the observed co-morbidity of sarcopenia and bone metabolism disorders [192]. Additionally, the expression of irisin is decreased in a mouse model of renal failure, and this may contribute to cortical bone loss [195]. Thus, irisin can strengthen the mineralization and mineral density of bone structure either directly or indirectly. The obesity pandemic nowadays often leads to obesity-related chronic kidney disease with microalbuminuria and glomerular hypertrophy, due to endothelial cell lesions in the renal arteries and glomerular capillaries. Irisin reduces urinary albumin excretion in obese mice, restores the VEGF–NO axis and attenuates renal fibrosis [196].

Irisin also attracts attention due to its antioxidant and anti-inflammatory properties. Irisin protects the cardiovascular system by optimizing glucose metabolism, which can prevent metabolic syndrome and, thus, decreases the risk of cardiovascular diseases [192]. Irisin increases proliferation of human umbilical cord endothelial cells (HUVEC) via extracellular signal-regulated kinase (ERK) and decreases their high glucose-induced apoptosis [197]. The proangiogenic effect of irisin has also been observed [198]. In T2DM, elevated plasma irisin may antagonize pro-atherogenic endothelial damage and, thus, serves as a silencer of negative vascular effects in diabetic conditions [199]. In ApoE-deficient mice, irisin decreases atherosclerotic lesions via suppressing vascular inflammation and endothelial dysfunction and reduces the inflammatory gene expression in partial ligated carotid artery lesions [200]. The same study reports a decrease in the macrophage chemoattractant protein-1 (MCP-1), IL-6, vascular cell adhesion protein 1 (VCAM-1) and intercellular cell adhesion molecule-1 (ICAM-1) mRNA, as well as significantly lower serum levels of MCP-1, IL-6, VCAM-1 and ICAM-1 in irisin-treated mice. Irisin inhibited the inflammatory response to oxidized low-density lipoprotein (oxy-LDL) in HUVEC, which increased HUVEC viability and decreased their apoptosis [198]. The oxy-LDL-induced inflammation in HUVEC was suppressed due to downregulation of MCP-1, IL-6, VCAM-1 and ICAM-1 mRNA expression and weaker ROS/p38 MAPK/NF-κB signaling. Lower levels of irisin are connected to increased oxidative stress and inflammation in humans. In obese children with T2DM and metabolic syndrome, there is a negative correlation between the irisin in blood plasma and the levels of VCAM-1, ICAM-2 and MCP-1 [201].

Irisin protects against ischemia–reperfusion (I/R) triggered heart injury. In the I/R model of excised mouse hearts, irisin reduced the myocardial infarction size and improved the left ventricle function [202]. The influence of irisin on I/R heart injury is associated with enhanced activity of mitochondrial SOD2, which reduces I/R-induced oxidative stress.

Irisin protects against I/R-induced acute kidney injury as well. This effect depends mainly on the upregulation of glutathione peroxidase 4, which leads to anti-inflammatory and anti-oxidative effects and improved mitochondrial function [203]. Exogenous irisin applied after hepatic I/R relieved the inflammatory response and reduced necrosis and hepatocyte apoptosis in mice [204]. This improvement in liver function after I/R was achieved due to the inhibition of mitochondrial fission, increased mitochondrial biogenesis of the PPAR-γ co-activator 1α and expression of the mitochondrial transcription factor.

### 4.3. Irisin Effects on the Brain and Kidney

Irisin improves brain function and this is well documented for learning and memory [205]. Irisin, which is produced in the periphery, is present in cerebrospinal fluid (CSF) because it can cross the blood–brain barrier in mice and humans [206,207]. Irisin from CSF increases the expression of the brain-derived neurotrophic factor, mainly in the hippocampus [208]. The neuroprotective effects of irisin are reported in ischemia-induced brain injuries, as well as in neurodegenerative disorders and disease models, such as for aging and Alzheimer’s disease [206,208] and the references therein. Additionally, irisin can support mental health due to its anxiolytic and antidepressant effects [209]. The CNS functions are attenuated because irisin modulates neuroplasticity-related genes in the prefrontal cortex and hippocampus [209], the synaptic plasticity, and downregulates the production of inflammatory factors IL-6 and IL-1β that limit neuroinflammation, decrease Aβ protein and tau protein formation and improve insulin resistance [205]. Although the impact of irisin on brain function is only partly clear, it becomes more and more evident that this myokine supports the proper working of the brain neurons either directly or indirectly by optimizing the activity of the astrocytes and microglia [205].

The irisin-dependent decrease in ferroptosis (iron-dependent lipid peroxidation) and kidney injury in the caecal ligation and puncture operation mice model for sepsis is observed in parallel with a reduction in the iron content, malondialdehyde level and ROS production, and increased GSH [6]. Similarly, irisin reduces ROS and iron release and supports the mitochondrial function in LPS-stimulated HK-2 cells. These beneficial effects are linked to the activation of SIRT1 and the Nrf2 transcription factor [6]. In the same way, irisin can alleviate acute lung injury through ferroptosis resistance, which is related to the activation of the SIRT1 and Nrf2 signaling pathways [210].

In conclusion, irisin is proven as an antioxidant, anti-aging, anti-inflammatory and anti-atherosclerotic mediator that through diverse mechanisms protects cell viability and functions in the kidney, heart, brain and liver, as well as in the bone and fat tissues (Figure 1B).

## 5. Melatonin as a Putative Antioxidant

A vast number of studies during the last several decades have identified and implicated the tryptophan-derived pineal gland hormone melatonin in almost all physiological processes and diseases in a variety of species, including plants, unicellular organisms and vertebrates. Since its discovery at the end of the 1950s [211], the pleiotropic effects of melatonin have attracted so much attention that today one can hardly find a topic in the biomedical literature where this molecule has not been hypothesized to play a role [212].

As one of the putative non-enzymatic endogenous antioxidants in the human body, melatonin is able to penetrate cellular membranes easily due to its small size and lipophilic nature and can be found virtually in all cells [213]. Melatonin and some of its metabolites were shown to have free radical scavenging activity and, thus, are considered as endogenous antioxidants [214]. In addition, studies have reported the synergistic actions of melatonin with vitamins C and E [215].

Indirect antioxidative effects of the pineal hormone can be exerted by a receptor-mediated modulation of the signaling pathways. Melatonin and its metabolites are able to stimulate antioxidative enzymes, such as SOD, GPx and glutathione reductase [216], and inhibit several prooxidant enzymes [217]. The regulation of the gene expression of these enzymes is a result of the binding of melatonin to its receptors that influences the downstream messengers and transcription factors [218]. In hepatoma H4IIE cells, melatonin was shown to attenuate many of the H_2_O_2_-induced alterations in the MAPK and mTOR signaling pathways [219]. Melatonin treatment activated the AMPK and PGC1α signaling in doxorubicin-treated H9c2 cells, thus supporting cardiac function and mitochondrial homeostasis and alleviating oxidative stress and apoptosis [220], (Figure 1B).

Numerous studies have examined the effects of supplementation of the pineal hormone and report a plethora of beneficial effects in healthy people and patients, some of which may be attributed to its antioxidant properties. Melatonin supplementation has been recently introduced as an adjuvant therapy for the treatment of SARS-CoV-2 respiratory infection [221]. The safety of exogenous melatonin is currently beyond concern; in many countries it is sold without a prescription. Only limited adverse effects have been reported from lower doses of melatonin administration and mild to moderate adverse events resulting from higher doses [222,223]. Considering the circadian rhythm and the binding affinity of the hormone to other biomolecules, further studies and trials are necessary to clarify the safety profile of long-term melatonin uptake.

## 6. Reductive Stress: Too Good Is No Good

Precise regulation of the balance of pro-oxidant and antioxidant molecules and ions is the basis of cellular redox homeostasis. Excessive intake of pro-oxidants leads to a well-defined state of oxidative stress. Extreme prevalence of reducing equivalents, such as GSH and NAD(P)H, and concentrations of basic oxidants O_2_^•−^ and H_2_O_2_ that are too low, can also be harmful for cell metabolism and functions [2]. Nowadays, reductive stress is defined as a significant shift in the cellular redox balance towards the reduction state due to the accumulation of endogenous or exogenous antioxidants and their reducing equivalents, which are able to affect the cellular metabolism via different mechanisms. Pleiotropic signal pathways and regulations can be responsible for or involved in the harmful effects from the excessive consumption of reducing substances, including food additives and spices with antioxidant properties. They can be classified as:

1. The pendulum effect: the pro-oxidant effects of antioxidants [224]. It is observed that an excess of GSH triggers pro-pathogenic mitochondrial oxidation and homeostasis that leads to hypoxia-like conditions [225]. Reductive conditions downregulate the antioxidant cell capacity by inhibiting the expression of antioxidant enzymes and GSH via SIRT1- and Nrf2-dependent signaling [6], and this can be followed by oxidative stress. For example, reductive stress followed by oxidative stress is suggested as a common mechanism of metabolic syndrome induction in hyperglycemia [226]. Some studies have found a causal relationship between reductive stress and excessive accumulation of reducing equivalents NAD(P)H and oxidative stress due to increased mitochondrial ROS production [227];
2. The misfolding of proteins in the endoplasmic reticulum is a key organelle in maintaining proteostasis and the unfolded protein response [228]. For this reason, acute or chronic reductive stress could hamper neurogenesis via the activation of the pathogenic GSK3β/Tau cascade to induce protein aggregation in neuroblastoma cells [229];
3. Nutritional supplements and spices with antioxidant activity that poorly penetrate into the mitochondria create significantly different redox conditions in the intracellular compartments. They cannot prevent oxidative stress in the mitochondria and the induction of apoptosis because their influence is restricted to the cytosol [4];
4. ROS are regulatory molecules that participate in intracellular and cell-to-cell signaling [1,7]. Reductive stress inactivates these physiological mechanisms. In endothelial cell, ROS and RNS increase (Ca^2+^)i through the activation of the Ca^2+^ influx through the transient receptor potential channels due to modification of the specific cysteine residues or through the increased production of the second messengers [230]. Similarly, ROS alter the activity of voltage-gated Ca^2+^ channels and voltage-gated potassium channels in different tissues [230], and the references therein. The superoxide anion reacts rapidly with NO to produce another reactive nitrogen species, peroxynitrite, but this process decreases NO bioavailability and terminates NO-dependent regulation in vascular and non-vascular tissues. On the other hand, the elimination of the superoxide anion increases the half-life of NO and this could worsen disease states associated with NO overproduction, such as septic shock, inflammatory diseases, neurodegenerative diseases, DM, I/R injury, adult respiratory distress syndrome and allograft rejection [231];
5. The tumor-accelerating effect of dietary antioxidants. Antioxidants N-acetylcysteine and vitamin E increase lung cancer progression by reducing p53 expression, i.e., by disrupting the ROS-p53 regulatory axis [232]. Such effects are observed with the application of vitamins A, C and many other antioxidants that lower ROS and accelerate human malignant melanoma cell migration and metastasis [233]. Similarly, the suppression of RAC1, a member of the Rho family GTPases, inhibits the RAC1-activated ROS generation pathway that promotes metastatic colonization in gastric cancer [234].

In conclusion, moderate doses of dietary antioxidants are useful for prophylaxis, but for the treatment of cancer and other diseases, they are not always recommended because they can induce reductive stress and attenuate or damage mechanisms of cell protection and adaptive signaling. Additionally, they often leave almost unchanged the high generation rate and level of mitochondrial ROS.

## 7. Cyanotoxins as Pro-Oxidative and Pro-Inflammatory Substances from the Environment

Cyanoprokaryotes, also known as green-blue algae or cyanobacteria, are photosynthetic prokaryotes. They produce a wide variety of toxic compounds that can be consumed through drinking water and foods [235,236]. Environmental cyanotoxins become quite dangerous for animals and humans in periods when the amount of cyanobacteria mass increases vastly during cyanoblooms that can happen either in sweet or salt waters [237]. Cyanotoxins, classified by the target tissues and organs, are neurotoxins, hepatotoxins, dermatoxins and cytotoxins, while the mechanisms of their toxicity are diverse [238]. Part of their toxicity depends on increased ROS production and decreased cellular antioxidant capacity [238], and the references therein. The cyanotoxins cylindrospermopsin (CYN), β-*N*-methylamino-l-alanine (BMAA), microcystins and nodularins decrease cell redox potential and attenuate mitochondrial function, the main ROS producer, leading to decreased cell viability with intensive metabolism in the first line, the neurons [239,240,241,242]. CYN leads to oxidative stress, either directly or indirectly by reducing GSH formation [240]. Thus, cyanotoxins can lead to neuroinflammation, neurodegeneration and neuronal disorders, such as Alzheimer’s and Parkinson diseases [243,244].

The cyanotoxins lipopolysaccharides, microcystins, and CYN activate the immune system and can provoke gastrointestinal inflammation [245]. Microcystins significantly alter the mouse gut microbiome and induce dysbiosis [240]. CYN reduces the viability of human gastrointestinal epithelial cells in culture and increases the permeability of the intestinal epithelium [238,246]. Additionally, cyanotoxins are more dangerous when consumed in combination, as they facilitate the absorption of other toxins due to their inflammatory action on the intestinal border [247,248]. This results in a leaky intestine and an impaired microbiome that can activate the hypothalamic–pituitary–thyroid, thyroid–gut, brain–gut, gut–liver and other axes, and provoke an autoimmune response and disorders like Hashimoto thyroiditis [5]. In these cases, anti-inflammatory and antioxidant nutritional protocols rich in food supplements for liver detox and cleansing, and for strengthening the gut microbiome can be followed before or together with therapeutical treatments [5].

## 8. Conclusions

The optimal ratio of pro- and antioxidant molecules is important for proper cellular function. The reviewed data show that long lasting deviations from this redox status generate oxidative or reductive stress, which is responsible for inflammation, allergic and autoimmune reactions, and also contributes to aging. The vasculature is mostly affected, along with the internal organs such as the kidney, heart, brain and liver, as well as the bone and adipose tissues being directly or indirectly subjected to harmful influences via circulation. Physical exercises stimulate the secretion of irisin, which is revealed as a potent protector of the abovementioned organs and tissues. Together with melatonin it can support redox homeostasis. Finally, moderate use of antioxidants seems to be a good prophylaxis against low doses of some cyanotoxins, which may contaminate sea foods and drinking water.
